# Invariant Natural Killer T lymphocytes as natural sensors for microbes: a two-edged sword in liver diseases

**DOI:** 10.3389/fimmu.2025.1662906

**Published:** 2025-10-06

**Authors:** Michelangelo Bauwelz Gonzatti, Alexandre Castro Keller

**Affiliations:** ^1^ Department of Physiology, University of California, San Francisco, San Francisco, CA, United States; ^2^ Laboratory of Experimental Immunopathology, Department of Microbiology, Immunology and Parasitology, Escola Paulista de Medicina/UNIFESP, São Paulo, Brazil

**Keywords:** iNKT, liver, microbiota, hepatitis, gut

## Abstract

The liver is a complex immunological organ characterized by a dual blood supply from the hepatic artery and portal vein, which continuously exposes it to microbial and dietary antigens, as well as potential pathogens that gain access to the circulation. This characteristic renders the liver particularly susceptible to immune activation, which may disrupt hepatic homeostasis and promote inflammation, thereby contributing to the pathogenesis of various liver diseases. Invariant natural killer T (iNKT) cells, a subset of liver-resident T lymphocytes, act at the intersection of hepatic immune surveillance and inflammatory responses. These cells are capable of rapid activation in response to glycolipid antigens presented by CD1d molecules and a broad range of pro-inflammatory stimuli, including cytokines and damage-associated molecular patterns. Perturbations in the intestinal barrier or dysbiosis of the gut microbiota can exacerbate hepatic exposure to microbes and metabolites, amplifying inflammatory signaling within the liver microenvironment. Although mouse models do not fully capture the complexity and heterogeneity of human liver diseases, the conserved nature of iNKT cell responses across species makes them useful for study their potential roles in human pathology. Furthermore, the discovery of specific iNKT agonists with polarizing ability emerges as an alternative to modulate the inflammatory microenvironment and the progression of hepatic damage. Therefore, a comprehensive understanding of iNKT cell dynamics under both physiological and pathological conditions is essential for the development of targeted therapeutic strategies to prevent or mitigate inflammatory liver diseases.

## Introduction

### Invariant natural killer T lymphocytes

Since the initial identification of a subset of murine T cells co-expressing an invariant Vα14 T cell receptor (TCR) and the natural killer (NK) cell marker NK1.1, the term Natural Killer T cells (NKT) has been widely used to describe T lymphocytes that share surface markers with NK cells (NK1.1 mouse; CD161/CD56 human) (1). However, as our understanding of NKT biology has evolved, this broad definition has proven to be both oversimplified and imprecise. First, the NK1.1 molecule is not present in all mouse strains and is not exclusive to NKT cells since CD8^+^ T lymphocytes can express NK1.1 and other NK markers, specially upon cytokine stimulation. Second, despite CD1d dependency for development, only those expressing the iTCR respond to stimulation with α-galactosylceramide (αGC), a marine sponge-derived lipid, complexed to the CD1d molecule (2). Therefore, the NKT lymphocyte population comprises two distinct subsets: a predominant population expressing an invariant TCR (Vα14Jα18 paired with Vβ8.2; Vβ7 or Vβ2 in mice; Vα24Jα18 paired with Vβ11 in humans) known as invariant NKT (iNKT), NKT or Type I NKT cells; and a less abundant, heterogeneous population expressing diverse TCRs, referred to as Type II NKT or NKT-like cells (3). Although both subsets depend on CD1d-mediated selection during thymic development, they differ substantially in terms of antigen specificity, functional profiles, and immunoregulatory roles. This mini review focuses specifically on the multifaceted behavior of murine iNKT lymphocytes in experimental models of liver disease, and detailed information regarding Type II NKT cells is available elsewhere (4, 5).

Notably, the iTCR from both human and mouse iNKT cells exhibit cross-species reactivity to CD1d, underscoring an evolutionary conserved recognition mechanism (6). When murine CD1d is replaced by its human counterpart, the iNKT repertoire shifts toward the murine Vβ8 iNKT subset, which mirrors the human Vβ11 iNKT cells (7). Therefore, these findings support that, with appropriate proportional consideration, murine models can effectively approximate human iNKT responses and their consequences.

### iNKT lymphocytes: heterogeneous triggered effector cells

In mice, both conventional αβ T and iNKT cell precursors enter the thymic medulla as double-negative (DN) cells and undergo TCR-β chain rearrangement at the DN3 stage. At this point, iNKT cells diverge from the conventional T cell lineage due to positive selection by CD1d-expressing double-positive (DP) cortical thymocytes. TCR/CD1d engagement induces the expression of transcription factors such as Egr-2 and promyelocytic leukemia zinc finger (PLZF), and the signaling through members of the SLAM family of receptors, and cytokines, drives iNKT differentiation into three major subsets that migrate to the periphery: iNKT1, iNKT2, and iNKT17 (8–10). Notably, additional iNKT subsets have been identified in the periphery (iNKTfh; iNKT10); however, current evidence suggests that these populations are not generated during thymic development but instead arise in response to peripheral environmental cues (11–16).

In contrast to conventional T lymphocytes, which exit the thymus as naïve cells, iNKT cells undergo epigenetic modifications during maturation, including histone acetylation that establishes constitutive mRNA expression for *IFNG* and *IL4*. This epigenetic priming poises iNKT cells to rapidly produce these cytokines within two hours of *in vivo* TCR stimulation with the specific antigen α-galactosylceramide (αGC) (17, 18). In addition to IFN-γ and IL-4, iNKT cells also rapidly secrete TNF-α and IL-13, whereas the production of IL-2, IL-10, IL-17A, and GM-CSF is typically observed at later time points (19). Although the mechanisms underlying iNKT cell functional plasticity remain unclear, evidence indicates that the differences among iNKT subsets reflect variations in the levels of cytokine production rather than distinct cytokine repertoires (8). It is proposed that the strength and/or quality of the signaling provided by TCR/CD1d-antigen engagement, in combination with co-stimulatory cues, shapes the cytokine output of iNKT cells (20–23).Therefore, the immunological context in which iNKT activation occurs plays a critical role in determining their functional response and, by extension, their contribution to the pathogenesis or resolution of immune-mediated liver diseases.

In humans, the exploration of iNKT cell thymic development remains a challenging issue; however, a transcriptomic study showed that human iNKT cells exhibit a developmental trajectory similar to that observed in mice (24). In the human periphery, despite phenotypic differences, single-cell RNA-seq analysis of enriched Vα24^+^Vβ11^+^ blood iNKT cells supports the existence of three primary transcriptional signatures: Th1/17/NK-like, Th2-like, and naive precursor cells, which resemble those observed in mice (25).

### Sensing microbes

As microbes breach epithelial barriers and enter the host, their pathogen-associated molecular patterns (PAMPs) are first detected by pattern-recognition receptors (PRRs), most prominently Toll-like receptors (TLRs). Although iNKT cells can respond to TLR stimulation, they also take advantage of other mechanisms to recognize alterations in the natural microbiota. For example, Gram-negative, lipopolysaccharide (LPS)-negative bacteria, such as *Sphingomonas* spp. and *Borrelia burgdorferi*, elicit IFN-γ production by iNKT cells through cognate recognition of foreign glycolipid antigens. *Sphingomonas* spp are rich in monoglycosylceramides, while *B. burgdorferi* expresses a monogalactosyl diacylglycerol (BbGL-II) (26–29). In contrast, LPS-positive bacteria engage iNKT cells through distinct pathways. For example, *Salmonella typhimurium*, requires interleukin-12 receptor (IL-12R) signaling, along with presentation of endogenous self-lipid antigens by CD1d molecules, to elicit an iNKT cell response. Conversely, during *Escherichia coli* infection, the synergistic action of IL-12 and IL-18 is sufficient to bypass TCR-mediated activation (28, 30, 31).

In addition to responding to pathogenic microorganisms, iNKT cells are also sensitive to alterations in the composition and activity of the commensal gut microbiota (32). For instance, both the distribution and cytokine profiles of iNKT cells in C57BL/6 mice have been shown to vary depending on the breeding facility of origin. This phenomenon disappears following co-housing, indicating a microbiota-dependent effect (33). Moreover, microbial colonization early in life shapes the pool of mucosal iNKT cells through a tolerogenic pattern, with lasting effects that prevent the development of exacerbated inflammatory responses (32). Although the precise mechanisms underlying this process remain incompletely understood, emerging evidence suggests a partial involvement of epigenetic regulation, specifically the suppression of *CXCL16*, a chemokine implicated in iNKT cell recruitment during liver inflammation (34, 35).

The healthy gut bacterial microbiota is primarily composed of *Firmicutes*, and to a greater extent, *Bacteroidetes* (25-50%). *Bacteroidetes* is a Gram-negative, LPS-positive phylum enriched in species capable of producing sphingolipids. Among these, *Bacteroides fragilis* is particularly notable for synthesizing αGC variants that modulate intestinal iNKT cell populations and promote a tolerogenic environment by inducing the production of IL-10 and IL-13, and to a lesser extent, IFN-γ (36–38). In contrast, the absence of Gram-positive bacteria has been associated with the accumulation of iNKT cells in the liver, particularly those biased toward IFN-γ production, suggesting that these commensals may play a role in suppressing exacerbated pro-inflammatory responses (39). Collectively, these findings indicate that gut bacterial microbiota modulate iNKT cell activity through distinct pathways. Given the importance of the gut-liver axis, such interactions may underlie the divergent roles of iNKT cells in liver physiology and disease.

### Microbes, iNKT, and the development of liver diseases

The gut microbiome has been implicated in the pathogenesis of several liver diseases, including nonalcoholic fatty liver disease (NAFLD) and autoimmune hepatitis (AIH) (40). NAFLD, recently reclassified as metabolic dysfunction-associated steatotic liver disease (MASLD), is the most prevalent chronic liver disease worldwide. MASLD is a multifactorial, systemic disease characterized by liver lipid accumulation (fatty liver), gut microbiota dysbiosis, and persistent low-grade inflammation. It affects approximately 25% of the global population and constitutes a significant risk factor for the development of liver fibrosis, cirrhosis, hepatocellular carcinoma (HCC), and liver-related mortality (41–44).

iNKT cells represent a major immune cell population in the mouse liver, comprising 5–40% of total leukocytes across different strains, and have been shown to play roles in both injury and repair mechanisms in models of liver injury (45–47). Gut and intrahepatic microbiomes can influence iNKT cell function in the liver through TCR-dependent and -independent mechanisms (48, 49). This positions iNKT cells as pivotal sensors of microbial diversity and microbiota status, capable of orchestrating immune responses across various liver diseases, while also making them susceptible to inappropriate responses depending on their activation context.

### Microbes, iNKT, and metabolic dysfunction-associated steatotic liver disease

Numerous studies have demonstrated a strong correlation between gut microbial composition and MASLD pathogenesis, as well as its progression to metabolic dysfunction-associated steatohepatitis (MASH), cirrhosis, and HCC (50–53). Supporting this association, germ-free (GF) mice are resistant to high-fat diet-induced obesity, a resistance that is reversed upon adoptive transfer of microbiota from obese donors (54, 55). Furthermore, antibiotic-treated obese mice exhibited improved glycemic control and reduced long-term obesity, reinforcing the role of the gut microbiota in liver physiology and metabolic regulation (55).

Intrahepatic immune profiling of NAFLD/MASLD patients revealed an accumulation of IFN-γ and IL-4-producing iNKT cells, with cytokine production levels positively correlating with disease activity scores (56). Similarly, a separate study comparing patients with simple steatosis and those with steatohepatitis found that hepatic iNKT cells in the latter group were more activated and produced higher levels of IFN-γ (57). Notably, an activated iNKT phenotype is also observed in the peripheral blood of NAFLD/MASLD patients (58). In parallel to the accumulation of activated hepatic iNKT cells, CD1d expression in liver-infiltrating mononuclear cells increases with disease severity, suggesting enhanced presentation of lipid antigens (56). Although mouse models of MASLD do not fully replicate all human clinical features, necessitating caution when extrapolating results, they remain valuable for exploring specific disease aspects, such as the relationship between iNKT cells and NAFLD-fibrosis (59, 60). For example, in the methionine/choline-deficient (MCD) diet model, iNKT-deficient animals (Jα18^-/-^) develop less severe liver injury than the wild-type group, supporting a pathological role of iNKT cells (61). In contrast, in high-fat diet models, the depletion of hepatic iNKT, in an IL-12-dependent manner, has been associated with the development of hepatosteatosis, suggesting a protective role for these cells (62). Although the mechanisms leading to iNKT depletion remain not fully understood, antibiotic treatment affects disease development. While disruption in microbial diversity and relative abundance of species exacerbate the diet-induced liver alterations, a broader treatment, targeting both Gram-positive and negative bacteria, prevents or attenuates NAFLD development (63–65). Given that iNKT cells can respond to microbial antigens and experimental NAFLD/MASLD is associated with gut microbiota alterations, it is plausible, but largely speculative, to imagine a link between dysbiosis, iNKT cells, and disease progression. Although NAFLD/MASLD patients show, in addition to iNKT activation, alterations in gut microbiota composition and metabolites profile, microbial signatures can overlap between MASLD and other metabolic disorders and are influenced by factors such as geography, ethnicity, sequencing methodology, and disease stage (66, 67). Unfortunately, the absence of a refined experimental model limits the ability to define clear cause-and-effect relationships between microbial species and disease development.

On the other hand, a positive correlation between probiotics, iNKT, and the attenuation of liver steatosis and metabolic disorders seems clearer. Meta-analysis and extensive review studies support the notion that pre/probiotic supplementation improves metabolic dysfunction-associated steatotic liver disease and decreases the risk for MASLD (68–71). Notably, the use of probiotic supplementation in experimental models of MASLD/MASH supports the positive relationship between the modulation of microbiota and resistance to MASLD-to-MASH progression (72, 73). Experimental models associated the protective effect of probiotic administration with an increase in hepatic iNKT cells. In high-fat diet-induced obese mice, probiotic supplementation (*Bifidobacteria, Lactobacilli*, and *Streptococcus thermophilus*) reduced weight and improved insulin resistance and steatosis (74). The number of hepatic, but not splenic, iNKT cells decreases within a week of initiating a high-fat diet, and before the development of insulin resistance and steatosis, suggesting that lipid overload induces iNKT cell death. Adoptive transfer of total NKT cells to iNKT-depleted obese mice improved obesity, insulin resistance, and steatosis, underscoring the critical role of these cells in controlling MASLD. Probiotic treatment reduced weight and improved both insulin resistance and steatosis in wild-type mice, while in CD1d^-/-^ obese mice these effects were not significant, indicating that the protective effects provided by probiotic supplementation are mediated by iNKT cell responses, though a contribution of NKT-II lymphocytes cannot be discarded (74). Given that improvements in obesity and hepatic steatosis were dependent on probiotic doses and correlated with an increased frequency of hepatic iNKT cells, it is possible to imagine a correlation between antigens and iNKT cell response. In line with this idea, lipids extracted from the *Bifidobacteria, Lactobacilli*, and *S. thermophilus* mixture activate and promote the proliferation of iNKT cells, which may explain how probiotic treatment prevents their depletion in obese mice (75). Collectively, these findings support the notion that the protective effects of probiotics against experimental MASLD/MASH are, at least in part, mediated by iNKT cell responses to microbial antigens.

### Microbes, iNKT, and primary biliary cholangitis

Primary biliary cholangitis (PBC), previously described as primary biliary cirrhosis, is an autoimmune condition where deregulated immune responses lead to a gradual destruction of intrahepatic bile ducts, marked by periportal inflammation and cholestasis, which can progress to cirrhosis. Like other autoimmune diseases, the presence of disease-specific autoantibodies and autoreactive CD4^+^ and CD8^+^ T lymphocytes are detected in PBC patients, where the immunodominant target is the mitochondrial pyruvirate dehydoxigenase complex (PDC-E2) (76–78). Alongside genetic susceptibility, environmental factors, such as bacterial infection and xenobiotics, have been proposed as environmental factors associated with tolerance breakdown (79). In this context, *Novosphingobium aromaticivorans*, a ubiquitous xenobiotic-metabolizing bacterium, is of special interest. First, this bacterium produces lipoylated proteins with a high degree of homology with the human PDC-E2, reacting with sera from almost 100% of anti-PDC-E2 positive patients (80). Also, as other members of the *Sphingomonas* genus, this bacterium expresses α-glycuronosylceramides in its cell wall, which is specifically recognized by iNKT cells (81). Patients with primary biliary cirrhosis (PBC) exhibit an increased frequency of iNKT cells in the liver compared to healthy controls, along with elevated CD1d expression in the epithelial cells of small bile ducts (82, 83). Furthermore, the exacerbation of PBC-associated fibrosis has been associated with increased levels of IL-17A in serum, which is proposed to be due to an enrichment of IL-17A-producing iNKT cells in the peripheral blood of PBC patients (84).

Experimental infection with *N. aromaticivorans* mimics the development of human primary biliary cirrhosis (PBC), in an iNKT-dependent manner. While CD1d^-/-^ mice are protected from liver injury following infection with *N. aromaticivorans*, the Vα14Tg mice, which overexpress Vα14Vβ8.2 iNKT cells, developed a more severe disease, indicating a pathological role for iNKT cells in biliary cirrhosis (81). Following infection, mice developed a long-lasting humoral response against both mammalian and microbial PDC-E2. Although bacterial burden was controlled within eight weeks, the animals progressively exhibited liver enlargement characterized by massive portal inflammation, severe bile duct damage and granuloma formation. The persistence of autoantibodies and a sustained inflammation in the absence of bacteria indicated an autoimmune origin of the liver pathology, closely mirroring the human disease. Notably, CD1d^-/-^ mice failed to mount a robust IgG response against PDC-E2, which was dependent on cognate interactions between iNKT cells and autoreactive plasmocytes, and were consequently protected from chronic liver inflammation. Adoptive transfer experiments demonstrated that disease could be reproduced injecting splenocytes from infected mice into both CD1d^+/+^ and CD1d^-/-^ recipients, with CD4 and CD8 T lymphocytes serving as the principal effectors rather than iNKT cells. Nonetheless, iNKT cells appear to play a pivotal initiating role: their rapid release of Th1 and Th2 cytokines enhances the activity of different arms of the immune system, thereby contributing to the breakdown of immunological tolerance that underlies autoimmune pathology (85, 86). Considering the evolutionary conservation of iNKT cells response, this experimental model supports the idea that cognate recognition of bacterial antigens by iNKT cells enhances the adaptive immune response against *N. aromaticivorans*. This heightened inflammatory environment, combined with antigen mimicry, likely disrupts peripheral tolerance and drives the development of autoimmune PBC.

### Microbes, iNKT, and hepatocellular carcinoma

The progression from MASLD to HCC involves a bidirectional relationship, where the host genetics influence the gut microbiome, which in turn can drive host epigenetic changes that promote disease progression (53). One of the proposed mechanisms for the progression of HCC is the metabolism of bile acids (BAs). The liver regulates circulating cholesterol levels by converting it into BAs, which are then metabolized in the intestine by the microbiota. Secondary BAs species generated by intestinal bacteria exert diverse physiological functions, including the inhibition of pro-inflammatory iNKT cells through the orphan nuclear receptor farnesoid X (FXR; NR1H4) (87–89). In this context, inhibition of secondary BAs metabolites by vancomycin treatment induces the production of CXCL16 by hepatic sinusoidal endothelial cells, and consequently, the accumulation of iNKT cells in the liver, which in turn controls the HCC burden (39). Vancomycin treatment depleted *Clostridium*, which is a Gram-positive bacterium responsible for producing secondary BAs via 7α-dehydroxylation (*Clostridium* cluster XIV) (90). Notably, depletion of *Clostridium* inhibited tumor metastasis specifically in the liver, indicating that it is the primary target for secondary BAs. Indeed, susceptibility to carcinogen-induced HCC in obese mice has been associated with increased levels of systemic deoxycholic acid (DCA), the metabolite resulting from the 7α-dehydroxylation reaction (91). Therefore, although *Clostridium* is part of the healthy commensal gut microbiota, dysbiosis may promote its overgrowth, leading to pathological outcomes by impairing the hepatic iNKT cell activity. Although the direct impact of microbial metabolites on iNKT cell responses in humans has not been clearly demonstrated, patients with HCC show a shift in iNKT cell subpopulations, characterized by an accumulation of CD4^+^ iNKT cells with a Th2-skewed cytokine profile and reduced cytotoxic potential (92). This phenotypic alteration may contribute to impaired anti-tumor immunity and facilitate disease progression.

### Microbes, iNKT, and autoimmune hepatitis

Autoimmune hepatitis (AIH) is a complex chronic inflammatory liver disease, and though the etiology remains unclear, several studies highlight the importance of microbiota and metabolites in AIH pathogenesis (93). This idea is strengthened by experimental models, such as concanavalin A (ConA)-induced AIH, where both iNKT cells and gut microbiota play a principal role (94). Although ConA triggers T-dependent hepatitis through direct interaction with the TCR, both CXCR6^-/-^ and Jα18^-/-^ mice are resistant to liver damage, indicating a pathological role for iNKT cells in ConA-induced AIH (34, 95, 96). In response to ConA, iNKT cells produce IL-4, which acts in an autocrine manner and increases the expression of granzyme B and Fas ligand (FasL) and, thereby, promotes direct hepatocyte injury. Supporting this, ConA fails to induce liver damage in Perforin^-/-^ or FasL-mutant^gld/gld^ mice (96). While ConA administration fails to induce liver injury in GF mice due to impaired iNKT cell response, the iNKT cells from GF and specific pathogen-free (SPF) animals react with the same intensity to αGC stimulation and induce similar hepatic injury in both mice. Therefore, gut microbiota is essential to elicit liver damage following ConA administration, reinforcing the role of iNKT in the pathology of ConA-induced AIH (97). Antibiotic treatment before ConA administration reduced glycolipid/CD1d complex levels in both portal blood lymphocytes and intrahepatic leukocytes, suggesting that most of the antigenic lipids found in the liver following ConA challenge originate from intestinal bacteria. Indeed, the levels of glycolipid/CD1d complexes increase in antibiotic-treated mice after oral administration of a heat-killed intestinal bacteria mixture upon ConA injection. Considering that ConA increases intestinal permeability, these data support a relationship between gut leak, AIH, and the activation of iNKT cells (97, 98). Although the precise mechanisms driving gut-liver-induced AIH remain to be clarified, ConA administration induces, along with changes in gut permeability, alterations in gut microbial content (99). Compared to steady-state controls, ConA-treated mice presented with lower 16S rRNA gene copy numbers for *Bifidobacterium* and *Lactobacillus* and higher copy numbers for *Enterobacteriaceae, a* family of diverse Gram-negative bacteria that includes pathogenic ones. Treatment with gentamicin, a potent broad-spectrum antibiotic particularly potent against members of the Enterobacteriaceae family, before ConA injection restrains liver injury, suggesting that ConA-induced gut dysbiosis, together with gut epithelial damage, provides the signals to iNKT-mediated AIH (99, 100). On the other hand, microbiota management sheds light on the relationship between bacteria and AIH, emerging as an alternative to control iNKT-mediated liver damage.

Gentamicin, but not vancomycin, treatment increases the abundance of *Bacteroides* spp within gut microbiota, with special attention to *B. acidifaciens*. Gentamicin alleviated ConA-induced liver damage by reducing CD95 (Fas) expression on the surface of hepatocytes, a phenotype reproduced by reconstituting a mouse with antibiotic-depleted gut microbiota with *B. acidifaciens* (101). Gentamicin did not impair the production of pro-inflammatory cytokines such as IFN-γ and TNF-α; thus, it is likely that *B. acidifaciens* exerts its hepatoprotective effect by modulating the interaction between iNKT cells and hepatocytes via the CD95L/CD95 pathway. Consistent with the beneficial effects of antibiotic-mediated gut microbiota manipulation on AIH, berberine, a traditional antibacterial agent, attenuates ConA-induced AIH by modulating gut microbiota composition and thereby improving the gut epithelial barrier and preventing liver inflammation (102). In addition to the indirect effects of microbiota modulation in iNKT cell-mediated AIH, it has been proposed that gut microbes stimulate peripheral dopamine production, which, in turn, suppresses iNKT cell activation (103).

Dopamine is a neurotransmitter with immunoregulatory activity over immune cells via stimulatory D1-like dopamine receptors (DR), DRD1 and DRD5, or inhibitory D2-like DR, DRD2, DRD3, and DRD4. Tyrosine hydroxylase (TH) catalyzes tyrosine into L-DOPA, which is rapidly converted to dopamine by aromatic L-amino acid decarboxylase (AADC). Besides dopaminergic neurons, dopamine can also be produced by other cell types and peripheral organs, as the kidney or adrenal medulla, and most of the peripheral dopamine is conjugated as sulfates or glucuronides, which are biologically inactive (104). In this context, the gut compartment appears as an essential component in the regulation of peripheral bioactive dopamine. First, by producing dopamine sulfate as a detoxification mechanism to prevent unwanted effects from high levels of circulating dopamine (105, 106). Second, by providing a bacterial diversity that can generate free dopamine by reversing the glucuronidation process that commonly occurs in the gut and produce different neurotransmitters, including dopamine (107–109). Dopamine plays a regulatory role in immune response and inflammatory reactions; therefore, it is reasonable to assume that alterations in gut composition may affect the levels of bioactive dopamine and compromise immune system homeostasis (104, 110, 111).

Despite several pieces of evidence suggesting that dopamine influences conventional T lymphocytes, little is known about its effects on iNKT cell behavior (112). Considering that hepatic murine iNKT cells express *drd1*, *drd3*, *drd4*, and *drd3* genes, it is correct to suppose that they must respond to dopamine stimulation (103). Indeed, the production of IL-4 and IFN-γ by hepatic iNKT cells upon stimulation with αGC is impaired in a dose-dependent manner by dopamine. Further analysis demonstrated that dopamine also inhibited cytokine production at the transcriptional level, and that this effect was dependent on the DRD1 signaling. Corroborating the inhibitory effect of dopamine on iNKT cell activity, the *in vivo* administration of A68930, a DRD1 agonist, inhibited ConA-induced AIH. Antibiotic treatment reduced the levels of TH in the small intestines, a phenomenon reversed by co-housing with control (non-treated mice), suggesting that gut microbes promote the local synthesis of dopamine. Bacterial clearance resulted in exacerbated iNKT cell responses to ConA, leading to more severe hepatic damage. Co-housing restored both intestinal TH levels and regulation of iNKT cell response to ConA. Moreover, pharmacological stimulation of DDR1 mimicked the microbiota’s ability to control ConA-induced AIH (103). Therefore, these findings demonstrate that gut microbes regulate peripheral dopamine levels and thereby restrain the magnitude of hepatic iNKT cells responses.

### Manipulating the inflammatory environment: iNKT-based immunotherapy

The first immunomodulatory effect attributed to iNKT cells was tumor growth suppression through IL-12-dependent stimulation (1). The subsequent discovery of αGC encouraged efforts to harness αGC/iNKT-based immunotherapy against cancer. Notably, beyond induction of IFN-γ and TNF-α, αGC stimulation also results in the production of Th2-prone cytokines and, consequently, expanded the interest through the immunomodulatory potential of iNKT cells. Structural modification of αGC molecule have yielded different analogues with biased responses. Introducing an aromatic group into the fatty acyl chain of αGC enhanced IFN-γ, over IL-4 production, indicating a potential use against tumors (113). In contrast, analogues with a shortened sphingosine moiety induce a shift through a Th2-like response with therapeutic potential in autoimmunity (114, 115). In this sense, natural healthy microbiota is a rich source of iNKT ligands with immunoregulatory properties (116, 117). Defining how distinct iNKT subsets contribute to liver disease is therefore critical for developing strategies to reshape the inflammatory milieu.

## 2 Concluding remarks

The relationship between iNKT and gut microbiome diversity under steady-state conditions remains a topic of debate, as conflicting results may result from differences in mouse strains or breeding environments (118, 119). Another critical but underexplored issue is whether the liver harbors a stable or transient microbiome, and how this microbial presence influences hepatic immune populations, particularly the iNKT cells (48, 120).

**Figure 1:**
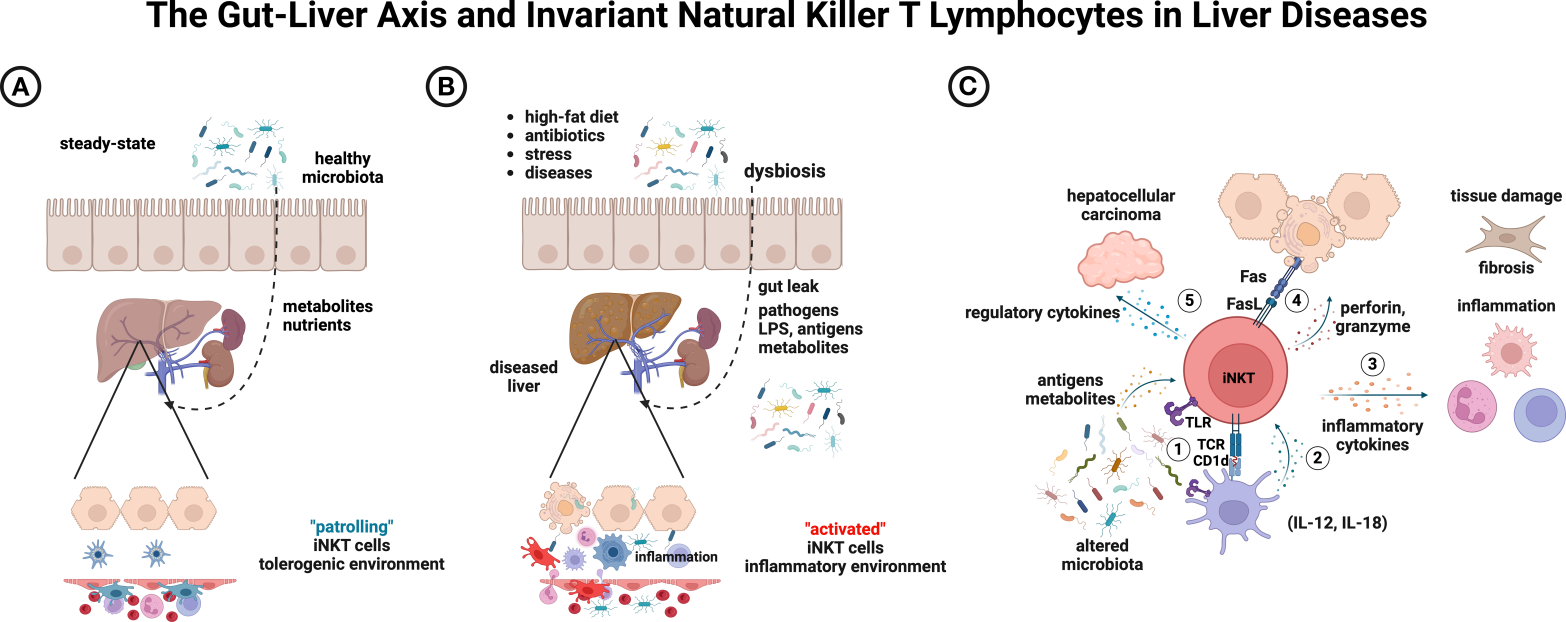
Conceptual model of interactions between iNKT lymphocytes and the gut microbiota, and their potential outcomes in liver diseases. **(A)** Under steady-state conditions, resident hepatic iNKT cell patrol along the sinusoidal endothelium. **(B)** Environmental factors may induce dysbiosis and impair gut integrity, leading to inflammation. **(C)** Altered microbiota can result in several outcomes: (1) direct iNKT engagement via LPS/TLR or cognate antigens; (2) indirect iNKT activation through IL-12/IL-18-producing cells; (3) secretion of pro-inflammatory cytokines that drive inflammation and fibrosis; (4) hepatocytotoxicity mediated by perforin/granzyme or Fas/FasL pathways; and (5) a regulatory shift that promotes hepatocellular carcinoma progression. Notably, probiotics or polarizing iNKT agonists can sustain a healthy microbiota, resolve inflammation, promote tissue repair, and enhance tumor control. Created with BioRender (https://biorender.com/ujlvmsp).

The iNKT–CD1d axis is evolutionarily conserved across species, and human iNKT cells retain the capacity to recognize bacterial-derived glycolipid antigens (29, 121, 122). While the role of iNKT cells in liver physiology remains incompletely understood, available data, primarily from studies on viral hepatitis, suggest their involvement in disease progression (123, 124). In chronically infected human livers, iNKT cells accumulate and exhibit a functional shift from an anti-tumoral, Th1-type response toward a pro-fibrotic, Th2-skewed profile. This phenotypic transition correlates with cirrhosis progression and is associated with increased hepatic CD1d expression, implicating a role for direct antigen recognition in the pathogenesis of chronic liver injury (125).The impact of the gut microbiome on the pathogenesis of immune-mediated liver diseases in humans is well established (126). Although the bidirectional interaction between iNKT cells and the commensal microbiome is not fully understood, iNKT cells are known to respond to both pathogenic and probiotic bacteria, resulting in distinct effects on liver physiology. Understanding the mechanisms that regulate the physiological and pathological roles of iNKT cells in the liver is crucial for developing novel therapeutic strategies for liver diseases (**Figure 1**).
